# Comparing human iPSC-cardiomyocytes *versus* HEK293T cells unveils disease-causing effects of Brugada mutation A735V of Na_V_1.5 sodium channels

**DOI:** 10.1038/s41598-019-47632-4

**Published:** 2019-08-01

**Authors:** Jeanne de la Roche, Paweorn Angsutararux, Henning Kempf, Montira Janan, Emiliano Bolesani, Stefan Thiemann, Daniel Wojciechowski, Michelle Coffee, Annika Franke, Kristin Schwanke, Andreas Leffler, Sudjit Luanpitpong, Surapol Issaragrisil, Martin Fischer, Robert Zweigerdt

**Affiliations:** 1 Institute for Neurophysiology, Hannover Medical School, Carl-Neuberg-Str.1, 30625 Hannover, Germany; 2 Siriraj Center of Excellence for Stem Cell Research (SiSCR), Faculty of Medicine, Siriraj Hospital, 2, Bangkoknoi, Bangkok, 10700 Thailand; 3 Department of Cardiac, Thoracic, Transplantation and Vascular Surgery (HTTG), Leibniz Research Laboratories for Biotechnology and Artificial Organs (LEBAO) and Hans Borst Zentrum (HBZ), Hannover Medical School, Carl-Neuberg-Str.1, 30625 Hannover, Germany; 4 Department of Stem Cell Discovery, Novo Nordisk A/S, 2760 Maaloev, Denmark; 5 Department of Anaesthesiology and Intensive Care, Hannover Medical School, Carl-Neuberg-Str.1, 30625 Hannover, Germany

**Keywords:** Ion transport, Molecular medicine, Induced pluripotent stem cells

## Abstract

Loss-of-function mutations of the *SCN5A* gene encoding for the sodium channel α-subunit Na_V_1.5 result in the autosomal dominant hereditary disease Brugada Syndrome (BrS) with a high risk of sudden cardiac death in the adult. We here engineered human induced pluripotent stem cell-derived cardiomyocytes (hiPSC-CMs) carrying the CRISPR/Cas9 introduced BrS-mutation p.A735V-Na_V_1.5 (g.2204C > T in exon 14 of *SCN5A*) as a novel model independent of patient´s genetic background. Recent studies raised concern regarding the use of hiPSC-CMs for studying adult-onset hereditary diseases due to cells’ immature phenotype. To tackle this concern, long-term cultivation of hiPSC-CMs on a stiff matrix (27–42 days) was applied to promote maturation. Patch clamp recordings of A735V mutated hiPSC-CMs revealed a substantially reduced upstroke velocity and sodium current density, a prominent rightward shift of the steady state activation curve and decelerated recovery from inactivation as compared to isogenic hiPSC-CMs controls. These observations were substantiated by a comparative study on mutant A735V-Na_V_1.5 channels heterologously expressed in HEK293T cells. In contrast to mutated hiPSC-CMs, a leftward shift of sodium channel inactivation was not observed in HEK293T, emphasizing the importance of investigating mechanisms of BrS in independent systems. Overall, our approach supports hiPSC-CMs’ relevance for investigating channelopathies in a dish.

## Introduction

Brugada Syndrome (BrS) is a rare autosomal dominantly inherited disease characterised by an ST-segment elevation in the right precordial leads of the electrocardiogram. Clinical symptoms include malignant arrhythmias, ventricular fibrillation and an increased risk of sudden cardiac death^1–4^. Up to 30% of BrS is caused by mutations in the *SCN5A* gene (BrS type 1) that encodes for the α-subunit of the sodium channel Na_V_1.5, which provides the main pathway for inward sodium currents in the heart ventricle mediating the rapid upstroke (phase 0) of the myocardial action potential (AP)^5^. More than 200 mutations of the *SCN5A* gene are known to be linked to BrS, the majority being missense mutations with heterozygous as well as homozygous gene inheritance^6–9^. A pathophysiological reduction of the Na_V_1.5 current density associated with a prominent transient outward potassium current (I_to_) is thought to cause the typical AP notch during early repolarization phase (phase 1) in myocardial cells. As the availability of human cardiomyocytes from patients is extremely limited, genetic modification of human pluripotent stem cells (hPSCs; including human embryonic and induced pluripotent stem cells) and the utilization of cardiomyocytes (CMs) derived thereof (hESC- or hiPSC-CMs) serves as an evolving technology to study physiological and pathophysiological functions of ion channels in human heart diseases^10–13^. However, due to the immature phenotype of early hPSC-CMs, recent studies raised concerns regarding the appropriateness of this approach^14–16^. For example, low levels of K_ir_ channel expression were found for hiPSC-CMs resulting in more depolarized resting membrane potentials poorly resembling the properties of native cardiomyocytes^17^. On the other hand, it has also been reported that long-term cultivation considerably increased K_ir_ channel current densities, even though channel expression remained low^18^. We here applied prolonged cultivation of hiPSC-CMs on a stiff glass matrix (>27 days post completion of differentiation), which has been shown to promote maturation of ventricular-like hESC-CMs^19^. Subsequently, extensive electrophysiological investigations of a disease-causing A735V-Na_V_1.5 mutation introduced into hiPSC-CMs were performed in comparison to both isogenic and non-genetically related hiPSC-CM controls (“wild type” WT) on the single cell level. Engineered hiPSC lines were generated by applying CRISPR/Cas9-based gene editing to induce a homozygous g.2204C > T point mutation into exon 14 of the *SCN5A* gene leading to an exchange of alanine to valine on the protein level (p.A735V). Amino acid A735 is located in the first transmembrane segment (S1) of domain II (DII) close to the first extracellular loop of the Na_V_1.5 protein. Notably, mutation A735V-related BrS induction was reported in four different clinical centres across Europe, America, and Japan^6^, thus representing a broad, potentially non-ethnicity restricted causative of the disease. Moreover, mutation A735V-Na_V_1.5 was previously correlated to a family of multiple affected individuals and shown to cause an electrophysiological BrS-phenotype according to a shift of the voltage dependence of activation when expressed as homozygous mutation in *Xenopus* oocytes system^8^. Here, to bridge the gap to such non-mammalian model, we also introduced the A735V-Na_V_1.5 mutation into another heterologous system that is HEK293T cells. This cell line is well established for investigating channelopathies and provides a relevant comparison to our hiPSC-CM approach. Combining these technologies, we present a novel hiPSC-CM disease model for A735V-Na_V_1.5 mutation-based BrS, revealing the causative effect of such point mutation irrespective of patient´s genetic background.

## Results

### Successful CRISPR/Cas9 mediated introduction of the A735V-Na_V_1.5 mutation in hiPSCs and differentiation into cardiomyocytes

**Figure 1:**
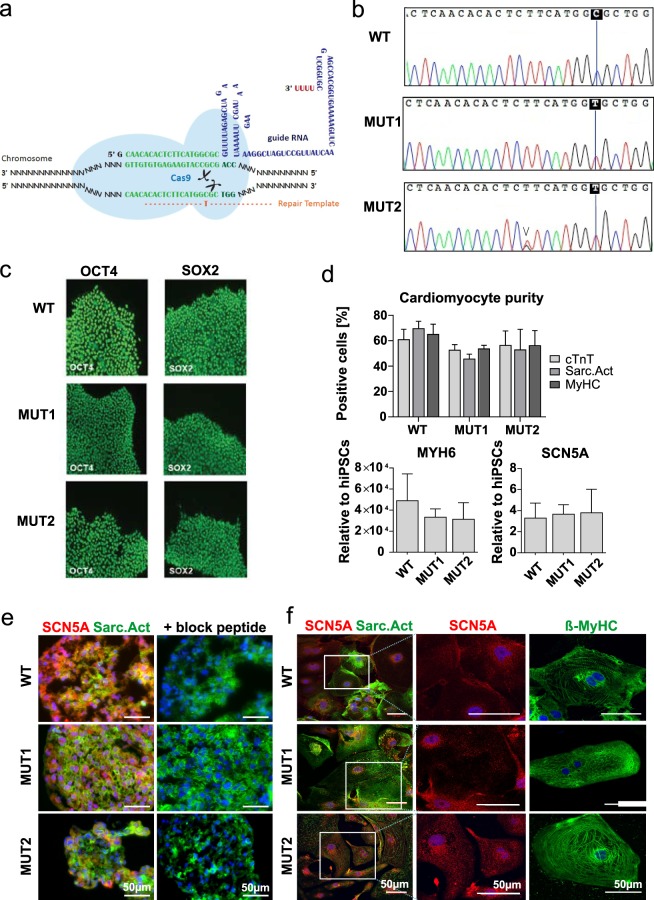
Inducing CRISPR/Cas9 mediated A735V-Na_V_1.5 mutation and cardiac differentiation. (**a**) Scheme of CRISPR/Cas9-mediated introduction of point mutation g.2204C > T in *SCN5A*. (**b**) Sequences of *SCN5A* showing mutation g.2204C > T in two derived MUT hiPSC-CMs compared to the isogenic WT hiPSC-CMs. One mutant clone (MUT2) possesses an additional heterozygote point mutation at position g.2197 T > G resulting in p.F733V and thus heterozygous mutant (the relevant sequence position is indicated by an arrowhead). However, this point mutation has not been reported in any cardiac disease and following Supplementary Fig. S7 mutation p.F733V presumably does not influence the channel properties. (**c**) Pluripotency markers (SOX2, OCT4) expression in WT and derived MUT hiPSC-CMs. (**d**) Flow cytometry for the CM-specific markers cardiac Troponin T (cTnT), sarcomeric Actinin (Sarc.Act) and pan-myosin heavy chain (MyHC) showed ~50–70% CMs for WT, MUT1 and MUT2 clones after 14 days of differentiation. Lower bar graphs show qRT-PCR results on *MYH6* and *SCN5A* expression levels for WT, MUT1 and MUT2 clones. (**e**) IF staining of cardiac aggregates with antibodies against *SCN5A* (red), sarcomeric actinin (Sarc.Act, green) and nuclei (DAPI, blue) suggesting robust Na_V_1.5 expression for WT, MUT1 and MUT2 cells, confirmed by a lack of *SCN5A* staining when adding the Na_V_1.5 block peptide. (**f**) Confocal images for IF staining of plated hiPSC-CMs (WT and the two A735V-Na_V_1.5 clones MUT1 and MUT2), after 29 days on glass coverslips. *Left panel:* IF staining specific to sarcomeric actinin (Sarc.Act, green) and *SCN5A* (red) revealing Na_V_1.5 expression in all three hiPSC-CM cell lines. *Middle panel:* corresponding magnification of framed sections in the left panel displaying a speckled Na_V_1.5 distribution pattern in hiPSC-CMs without obvious differences in cells from WT *versus* mutant clones. *Right panel:* IF staining for β-MyHC (green) showing organized sarcomere structures and matured cardiac phenotypes in all three hiPSC-CM cell lines. Scale bar for all three panels is 50 µm.

As schematically presented in Fig. 1a, a homozygous g.2204C > T mutation was engineered into the *SCN5A* locus encoding for a p.A735V mutation in Na_V_1.5. Specificity was confirmed by sequence analysis in two independently derived clones designated MUT1 and MUT2 (Fig. 1b). Immunofluorescence (IF) staining specific to OCT4 and SOX2 exemplarily revealed homogeneous expression of pluripotency-associated markers in representative MUT1/2 colonies (Fig. 1c) equivalent to the original isogenic hiPSC line (designated wild type; WT).

Directed cardiomyogenesis by chemical WNT pathway modulators^20,21^ enabled the induction of ~50–70% hiPSC-CMs content across all lines tested, as confirmed by flow cytometry for three independent markers: cardiac Troponin T (cTnT), sarcomeric Actinin (sarc.Act) and pan-myosin heavy chain (MyHC) (Fig. 1d). Equivalent cardiac differentiation of WT *versus* mutant clones was further confirmed by qRT-PCR for *MYH6* (α-MyHC isoform; Fig. 1d, lower left) and *SCN5A* expression (Fig. 1d, lower right), suggesting that the g.2204C > T mutation did not impact on *SCN5A* mRNA abundance. Moreover, IF staining of sections of cardiac aggregates (generated by suspension culture^20–22^) revealed Na_V_1.5 protein expression for both WT and mutant clones, whereby utilization of a *SCN5A* specific blocking peptide confirmed staining specificity (Fig. 1e). IF staining of cardiac aggregate-derived dissociated CMs cultured for 29 days on glass coverslips revealed (i) prominent expression of Na_V_1.5 comparable across WT and mutant hiPSC-CMs with speckled channel distribution patterns confirming previously published confocal images of Na_V_1.5 in neonatal^23^ and iPSC-derived cardiomyocytes^24,25^ (ii) prominent ß-MyHC isoform expression (encoded by *MYH7*; α- to ß-MyHC isotype switch being an accepted maturation mark in the native heart and in hPSC-derived CMs as well^19^) and (iii) induction of organized sarcomere structures and abundant CMs’ bi-nucleation (Fig. 1f).

### Mutant A735V-Na_V_1.5 reduces the AP upstroke velocity in hiPSC-CMs

**Figure 2:**
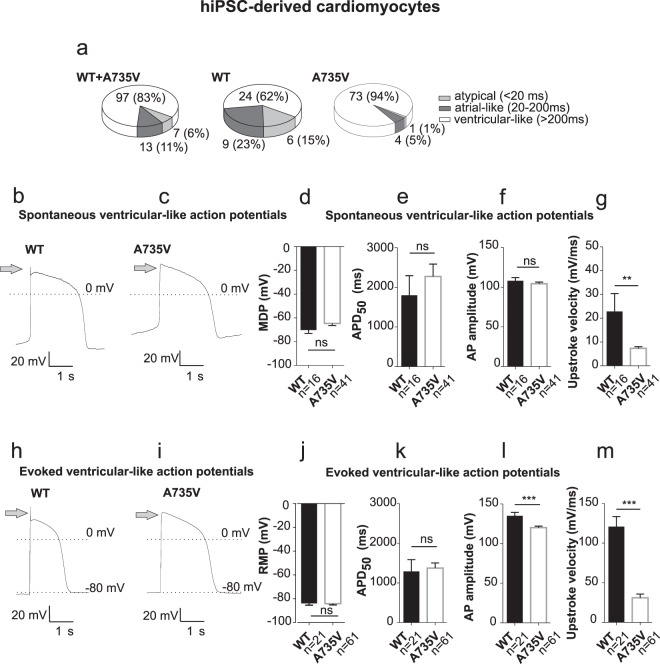
Mutant A735V-Na_V_1.5 channels reduce the upstroke velocity of APs in hiPSC-CMs. (**a**) Distribution of cardiomyocyte phenotypes for all hiPSC-CMs (WT + A735V, left), WT hiPSC-CMs (middle) and A735V hiPSC-CMs (right). Cells were classified according to their AP duration as ventricular-like (APD_50_> 200 ms), atrial-like (APD_50_: 20-200 ms) or atypical (APD_50_ <20 ms or no AP). (**b,c**) Representative traces of spontaneous action potentials from ventricular-like hiPSC-CMs expressing WT (**b**) or mutant A735V (**c**) Na_V_1.5 channels. (**d–g**) Electrophysiological properties of spontaneous APs (mean values ± s.e.m.) from WT (n = 16) and A735V hiPSC-CMs (n = 41): Maximum diastolic potential MDP (**d**), AP duration at 50% repolarization level APD_50_ (**e**), AP amplitude (**f**) and upstroke velocity (**g**). (**h,i**) Representative traces of evoked action potentials from ventricular-like hiPSC-CMs expressing WT (**h**) or mutant A735V (**i**) Na_V_1.5 channels. For such recordings CMs were hyperpolarized to physiological resting potentials (-80 mV) and action potentials were elicited by intracellular application of short depolarizing current pulses (≤3 nA, 1 ms). (**j–m**) Electrophysiological properties of evoked APs (mean values ± s.e.m.) from WT (n = 21) and A735V hiPSC-CMs (n = 61): Resting membrane potential RMP (**j**), AP duration at 50% repolarization level APD_50_ (**k**), AP amplitude (**l**) and upstroke velocity (**m**). Arrows in (**b,c,h,i**) mark the phase 0 upstroke of action potentials that is significantly decelerated in BrS-mutant A735V-Na_V_1.5 hiPSC-CMs.

Current clamp analyses of long-term plated CMs showed action potentials (APs) with a plateau phase of >200 ms (AP duration at 50% of their repolarization level (APD_50_) at RT) in ~83% of all CMs tested thus being classified as ventricular-like. The remaining ~17% were classified as atrial-like (APD_50_ 20–200 ms) or atypical CMs (APD_50_ < 20 ms or unexcitable CMs; Fig. 2a). Our further analyses focused on ventricular-like hiPSC-CMs since BrS is characterised by transmural electric gradient between the ventricular epi- and endocardium. 67% of WT and 56% of A735V mutated ventricular-like cells were spontaneously active. The residual cells (33% and 44%, respectively) remained silent but APs could be evoked by injections of short depolarizing current pulses (≤3 nA, 1 ms). Even though the maximum diastolic potential (MDP) of spontaneous APs in the mutant cells was insignificantly depolarized as compared to WT cells (Fig. 2d; WT: −70 ± 3 mV; A735V: −65 ± 2 mV; Student’s *t*-test, *p* = 0.0926), AP shapes were similar at a first glance (Fig. 2b,c). While APD_50_ values (Fig. 2e) (WT: 1802 ± 494 ms; A735V: 2274 ± 314 ms) and mean values for the AP amplitude (WT: 108 ± 4 mV; A735V: 104 ± 2 mV; Fig. 2f) were comparable, only APs of WT CMs displayed a sharp peak in phase 0 depolarization with subsequent phase 1 repolarization (grey arrow, Fig. 2b,c). Accordingly, the mean upstroke velocity of the spontaneous APs was significantly faster for WT (23 ± 7 mV/ms, n = 16) *versus* A735V CMs (7 ± 1 mV/ms, n = 41, Fig. 2g; Student’s *t*-test, *p* < 0.01).

To gain comparable conditions for WT and A735V CMs, hyperpolarizing holding currents were injected (range: 0 to −100 pA) to reach constant resting membrane potentials of about −80 mV equivalent to native ventricular cardiomyocytes. This approach mimics the contribution of an inwardly rectifying potassium channel (K_ir_) often less expressed in hiPSC-CMs^26^. Consequently, CMs generally stopped firing but action potentials could be evoked by intracellular stimulation (Fig. 2h,i). Again, the sharp peak in phase 0 was common in evoked APs of WT CMs but missing in A735V cells. Furthermore, the rapid repolarization phase 1 was non-visible in A735V CMs, probably corresponding to the lack of fast depolarization in the mutant (Fig. 2c,i). Even though the resting membrane potentials (RMPs) and APD_50_ values were comparable (Fig. 2j,k), recordings revealed a considerable decrease in AP amplitude (WT: 135 ± 4 mV; A735V: 120 ± 2 mV; Fig. 2l) and a prominent reduction in AP upstroke velocity by ~74% for A735V CMs (WT: 121 ± 13 mV/ms; A735V: 31 ± 5 mV/ms; Fig. 2m). It is worth emphasizing that hyperpolarization towards a consistent resting membrane potential generally leads to a faster upstroke of the evoked *versus* spontaneous APs for the same set of available sodium channels (Fig. 2m compared to Fig. 2g). However, the depolarization pattern of A735V CMs was decelerated for both conditions, strongly indicating altered activation or inactivation characteristics of the mutant sodium channels.

### Mutation A735V shifts the activation curve of Na_V_1.5 in hiPSC-CMs

**Figure 3:**
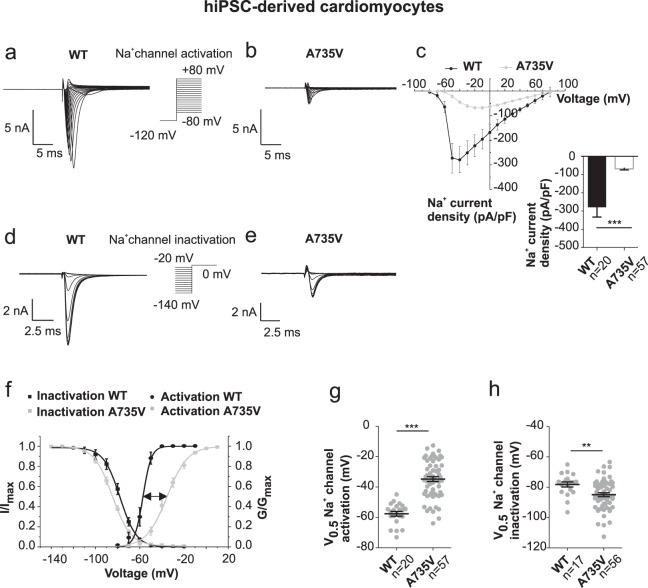
Mutation A735V shifts the activation curve of Na_V_1.5 channels in hiPSC-CMs. (**a,b**) Voltage protocol for sodium channel activation and representative current traces of cardiac Na_V_1.5 channels recorded from WT hiPSC-CMs (**a**) and mutant A735V hiPSC-CMs (**b**). (**c**) Voltage dependence of mean Na_V_1.5 current densities (pA/pF) from WT and mutant A735V hiPSC-CMs. Inset: Comparison of the maximum Na_V_1.5 current density from WT and mutant A735V hiPSC-CMs. (**d,e**) Voltage protocol for sodium channel inactivation and representative current traces of cardiac Na_V_1.5 channels recorded from WT hiPSC-CMs (**d**) and mutant A735V hiPSC-CMs (**e**). (**f**) Sodium channel activation (G/G_max_) and inactivation (I/I_max_) curves of Na_V_1.5 channels from WT and A735V hiPSC-CMs. Solid lines represent Boltzmann functions that were fit to the mean values. (**g,h**) Scatter plots and mean values (±s.e.m.) of mid voltages (V_0.5_) of activation (**g**) and inactivation (**h**) for WT and A735V-Na_V_1.5 channels from hiPSC-CMs. - Data volume for sodium channel activation: WT, n = 20; A735V, n = 57; for sodium channel inactivation: WT, n = 17; A735V, n = 56.

To directly investigate the currents underlying the AP upstroke whole-cell voltage clamp recordings were performed under the same ionic conditions (Fig. 3a,b). WT CMs produced prominent inward currents that were carried by sodium and calcium ions (Supplementary Fig. S1). The contribution of calcium currents was determined by starting from a pre-pulse potential of −40 mV (instead of −120 mV), where sodium channels are completely inactivated. Such calcium currents accounted for only 1–2% of the total current amplitude and were thus considered negligible for WT hiPSC-CMs (Supplementary Fig. S1). Notably, absolute Ca^++^ current amplitudes for WT and A735V CMs were comparable (Supplementary Fig. S1). Since sodium currents of mutant A735V cells were clearly reduced (Fig. 3b), the Ca^++^ current fraction of the total current amplitude was about 10% on average and has been subtracted from the recordings for mutant cells. Figure 3c displays the voltage dependence of mean sodium current densities for both WT and A735V CMs. WT CMs elicited their maximum current density (−279 ± 53 pA/pF) at −40 mV (Fig. 3a,c). In contrast, A735V CMs showed a strong and substantial reduction in mean current density by ~76% and reached maximum values at −10 mV (−68 ± 6 pA/pF), indicating a shift of the current-voltage dependence to more positive potentials (Fig. 3c). Relative conductance (G/G_max_) was obtained from the I-V relationship and plotted *versus* the test potential to reveal the sodium activation curve (Fig. 3f; circle symbols). A striking rightward shift of more than 20 mV was observed for the mutant A735V channel compared to WT. Figure 3g shows a scatterplot of voltages for half maximal activation (V_0.5_). Mean values of V_0.5_ were −58 ± 0.4 mV for WT and −35 ± 0.5 mV for A735V. Since amplitudes of sodium currents also depend on the relative proportions of channels in its resting and inactivated closed state, we additionally studied voltage dependence of sodium channel inactivation in WT and mutant hiPSC-CMs (Fig. 3d,e). Plotting the relative current amplitudes I/I_max_ at test pulse (0 mV) *versus* pre-pulse potentials (−140 mV to −20 mV) displays the inactivation curves for WT and mutant A735V channels (Fig. 3f, rectangular symbols). A small but significant leftward shift of the A735V inactivation curve was observed (WT: −77 ± 0.3 mV, A735V: −84 ± 0.1 mV; Fig. 3h, Student’s *t*-test, *p* < 0.01). This shift results in a reduced availability of resting sodium channels for the AP upstroke at physiological membrane potentials. While ~40% of WT sodium channels are inactivated at −80 mV, the fraction is even larger for A735V mutants, where> 60% of sodium channels occupy the inactivated state. Thus, both the rightward-shift of the activation curve and the leftward-shift of the inactivation curve of mutant Na_V_1.5 channels might contribute to reduced current amplitudes in phase 0 depolarization of hiPSC-CM action potentials. Notably, conclusions from shifts in activation and inactivation curves must be considered with care given the challenges of proper voltage control resulting from the large cell size of hiPSC-CMs and their large current amplitudes as well (see WT Na_V_1.5 in Fig. 3a). As shown below, such findings require further substantiation by experiments on well-clamped HEK293T cells.

### Effects by mutation A735V are highly robust and hiPSC clone-independent

**Figure 4:**
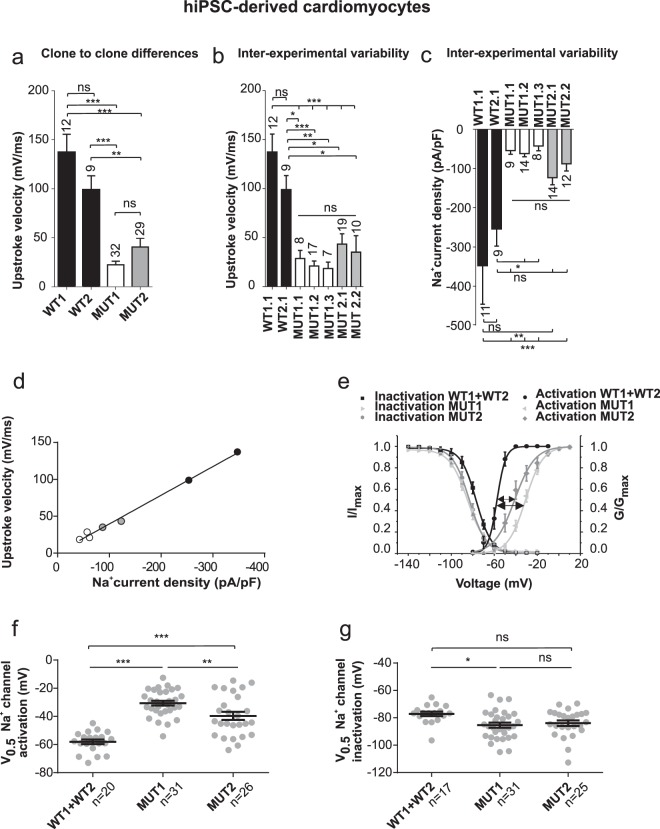
Effects of A735V mutation are reproducible from clone-to-clone and for different approaches of hiPSC-CMs. (**a**) Mean values (±s.e.m.) of upstroke velocities of two WT and two A735V mutant clones. There are no differences among WT clones or A735V clones, but differences between WT and A735V clones are highly significant (one way ANOVA with Tukey’s post-hoc comparison test F(3,78) = 26.32, *p* < 0.001). (**b**) Inter-experimental variability between different approaches with WT and mutant A735V hiPSC-CMs showing no changes in upstroke velocities among themselves, while WT *versus* A735V differed considerably from each other (one way ANOVA with Tukey’s post-hoc test F(6,75) = 12.82, *p* < 0.001). (**c**) Inter-experimental variability of mean maximal sodium current densities for WT and A735V mutant hiPSC-CMs (one way ANOVA with Tukey’s post-hoc test F(6,70) = 7.467, *p* < 0.001). (**d**) An increase in sodium channel density directly correlates to upstroke velocity acceleration of the same cells throughout the different hiPSC-CM approaches corroborating the effect of A735V sodium channel mutation on upstroke velocity (**d**, linear regression with R² = 0.9937 and F(1,5) = 749.9; n = 7-19). Coloured symbols represent mean values of different approaches as depicted in (**b,c**). (**e**) Sodium channel activation (G/G_max_) and inactivation (I/I_max_) curves of Na_V_1.5 channels from WT and two clones of A735V hiPSC-CMs. Solid lines represent fits with Boltzmann functions. (**f**) Mid-voltages V_0.5_ of sodium channel activation for two independent A735V clones as compared to WT hiPSC-CMs (one way ANOVA with Tukey’s post-hoc test F(2,74) = 37.76, *p* < 0.001). (**g**) Mid-voltages V_0.5_ of sodium channel inactivation for two independent A735V clones as compared to WT hiPSC-CMs (one way ANOVA with Tukey’s post-hoc test, F(2,70) = 4.261, *p* < 0.05). Data volume in panels (a–c) and (f–g) is as indicated by numbers. (e) comprises the same cell numbers as in (f) and (g).

Above results represent a compilation of analysing two independent clones for WT and A735V mutants, each investigated in 2–3 biological repeats. Since functional properties of hPSC-CMs may vary, we evaluated each clone separately (Fig. 4a,e–g) and investigated inter-experimental variants (Fig. 4b,c). Comparing the upstroke velocity, we noted that CMs from the two WT clones did not significantly differ (137 ± 18 mV/ms for WT1 as compared to 99 ± 14 mV/ms for WT2, Fig. 4a, Student’s *t*-test, *p* = 0.1379). This substantiates our parallel findings for both mutant A735V clones showing a substantial reduction in upstroke velocity (MUT1: 22 ± 4 mV/ms and MUT2: 41 ± 9 mV/ms) compared to WT CMs (one way ANOVA with Tukey’s post-hoc comparison test F(3,78) = 26.32, *p* < 0.001). Moreover, although upstroke velocity of WT2 was less prominent, it still differs significantly from the faster mean upstroke velocity of MUT2 (*p* < 0.01, Fig. 4a). Importantly, no substantial differences in these results were observed between five independent differentiation experiments with A735V CMs. In contrast, differences in upstroke velocities between WT and A735V cells were obvious in all individual experiments (Fig. 4b), which also applies to the comparison of maximum current densities (Fig. 4c, one way ANOVA with Tukey’s post-hoc test F(6,75) = 12.82, *p* < 0.001). An increase in sodium current density directly correlates to upstroke acceleration of the same cells thus corroborating the causative effect of the A735V sodium channel mutation on AP upstroke modification (Fig. 4d, linear regression R² = 0.9937 and F(1,5) = 749.9). Regarding the voltage dependence of activation, a shift towards more positive potentials for both A735V clones *versus* WT was found, whereby a difference between MUT1 and MUT2 was observed (Fig. 4e,f; WT1 + WT2: V_0.5_ −58 ± 2 mV; MUT1: V_0.5_ −31 ± 2 mV; MUT2: V_0.5_ −40 ± 3 mV). In contrast, no difference between the A735V clones was identified for the inactivation and only a slight difference between MUT1 compared to WT was found (Fig. 4e,g; WT1+WT2: V_0.5_ −77 ± 2 mV; MUT1: V_0.5_ −85 ± 2 mV; MUT2: V_0.5_ −84 ± 2 mV). Taken together, we have demonstrated substantial and reproducible shifts of activation curves for the A735V mutation, while the effect on voltage dependence of inactivation was less evident.

### Mutation A735V shifts the activation curve of Na_V_1.5 channels in HEK293T cells

**Figure 5:**
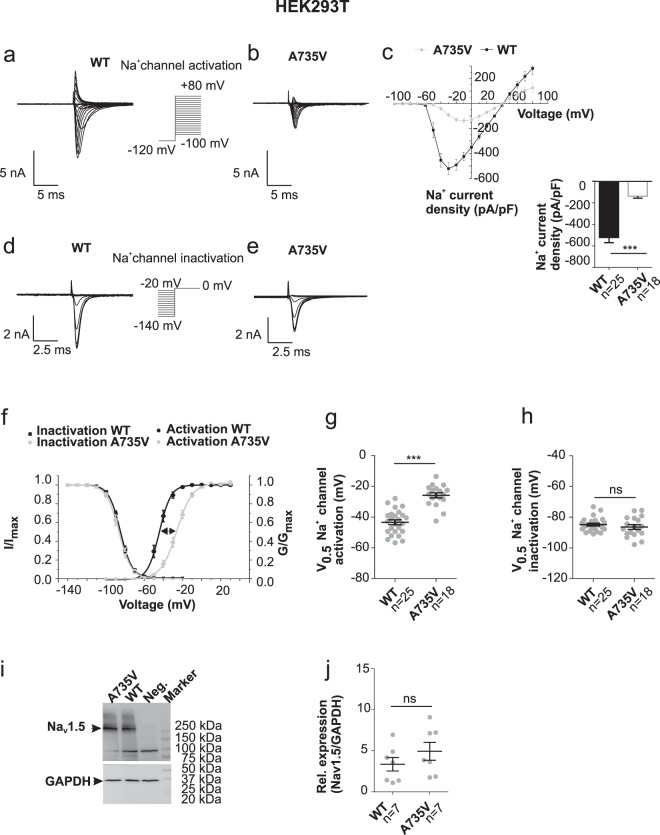
Mutation A735V shifts the activation curve of Na_V_1.5 channels heterologously expressed in HEK293T cells. (**a,b**) Voltage protocol for sodium channel activation and representative current traces of WT (**a**) and mutant A735V (**b**) -Na_V_1.5 channels heterologously expressed in HEK293T cells. **c**) Voltage dependence of mean current densities (pA/pF) from HEK293T cells expressing WT or mutant A735V-Na_V_1.5 channels. Inset: Bar graph on comparison of the maximum current density for WT and mutant A735V Na_V_1.5 channels. (**d,e**) Voltage protocol for sodium channel inactivation and representative current traces of WT (**d**) and mutant A735V (**e**) Na_V_1.5 channels heterologously expressed in HEK293T cells. (**f**) Sodium channel activation (G/G_max_) and inactivation (I/I_max_) curves of WT and A735V Na_V_1.5 channels expressed in HEK293T cells. Solid lines represent fits with Boltzmann functions. (**g,h**) Scatter plots and mean values (±s.e.m.) of mid-voltages (V_0.5_) of activation (**g**) and inactivation (**h**) for WT and mutant A735V-Na_V_1.5 channels. (**i**) Representative western blot analysis for heterologous Na_V_1.5 channel expression in HEK293T cells, showing similar intensities for WT and mutant A735V-Na_V_1.5 bands. Untransfected HEK293T cells served as negative control (Neg.). GAPDH is visualized as loading control for comparable concentrations of total protein. (**j**) Scatter plot and mean values (±s.e.m.) of relative expression levels of Na_V_1.5/GAPDH (WT: n = 7; A735V: n = 7, Student´s *t-*test, *p* = 0.27). - Data volume in (c) and (f–h): WT, n = 25; A735V, n = 18.

To reveal potential advantages but also to avoid pitfalls and prevent premature conclusions, the hiPSC-CMs data have to be compared to alternative assays. Accordingly, we used the established heterologous HEK293T expression system, which is ideally suited for patch clamping due to negligible endogenous sodium channels, small cell size and roundish appearance granting an effective voltage control over the entire cell membrane. Figures 5a,b show the voltage protocol and representative whole-cell patch clamp recordings for sodium currents from HEK293T cells transiently expressing WT (a) or mutant A735V-Na_V_1.5 channels (b). Representative current traces and the I-V plot depicted in Fig. 5c clearly demonstrate that mean amplitudes of mutant A735V currents were extensively reduced by about 77% (WT: −523 ± 48 pA/pF at −30 mV, n = 25; A735V: −139 ± 18 pA/pF at −10 mV, n = 18, Fig. 5c). Moreover, the voltage of maximum current density was shifted to less negative potentials in the mutant constructs, confirming the hiPSC-CM data. The rightward-shift of the I-V curve corresponds to a marked shift of the activation curve to more depolarized potentials (Fig. 5f,g, WT: V_0.5_ −44 ± 0.3 mV; A735V: V_0.5_ −26 ± 0.3 mV). Sodium channel inactivation was studied using pre-potentials between −140 and −20 mV and a subsequent test step to 0 mV (Fig. 5d,e). Even though current amplitudes were generally smaller for A735V, the inactivation curve perfectly overlapped with WT controls (Fig. 5f). Thus, in contrast to hiPSC-CMs, inactivation of Na_V_1.5 channels remained almost unchanged in the heterologous system (WT: V_0.5_ −85 ± 0.03 mV; A735V: V_0.5_ −87 ± 0.06 mV; Fig. 5h). We next asked whether the shift in the activation curve by mutation A735V is caused by improper voltage clamp conditions in HEK293T cells, when large WT currents were obtained under physiological sodium concentrations. Additional experiments with reduced external sodium concentration revealed current amplitudes in WT cells equivalent to the A735V-Na_V_1.5 mutant under physiological conditions (Supplementary Fig. S4). These experiments confirmed the relevance of the observed shift in the activation curve (Fig. 5f,g), since WT sodium channel properties remained unaffected of the sodium concentration variations, indicating proper voltage clamp conditions in HEK293T cells even at large current amplitudes.

Reduced sodium current amplitudes in mutant A735V (Fig. 5b,e) may result from lower expression levels. Notably, western blot analysis revealed no significant difference in the relative Na_V_1.5 protein expression between cells transfected with the same amount of cDNA for WT or A735V-Na_V_1.5 (Fig. 5i,j, Student’s *t*-test, *p* = 0.2701). Moreover, extracellular biotinylation of membrane proteins showed that the portion of sodium channels present in the outer cell membrane of HEK293T cells remained unchanged (or was even more pronounced) when expressing A735V mutant channels as compared to WT controls (Supplementary Fig. S5). These results were substantiated for hiPSC-CMs as well (Supplementary Fig. S6), even though respective biotinylation experiments of Na_V_1.5 channels in hiPSC-CMs are challenging, presumably due to the relative low abundance of Na_V_1.5 protein and potentially poor protein accessibility for antibody binding. In conclusion this supports the view that altered channel properties in the mutants (but no obvious changes in the channel expression and insertion into the outer cell membrane) are causative for the observed current reduction by Brugada mutation A735V. Here, the mutation-dependent changes in channel properties are documented by the shift of the activation curve, but may also include modifications in single channel conductance or absolute open probability.

In summary, these experiments revealed that mutation-dependent malfunction in Na_V_1.5 channel activation without reduction in membrane insertion can be monitored in both human iPSC-CMs and the human heterologous expression system. However, a specific alteration of channel inactivation was observed in hiPSC-CMs, only.

### Mutation A735V prolongs the time constant of sodium channel recovery in hiPSC-CMs and HEK293T cells

**Figure 6:**
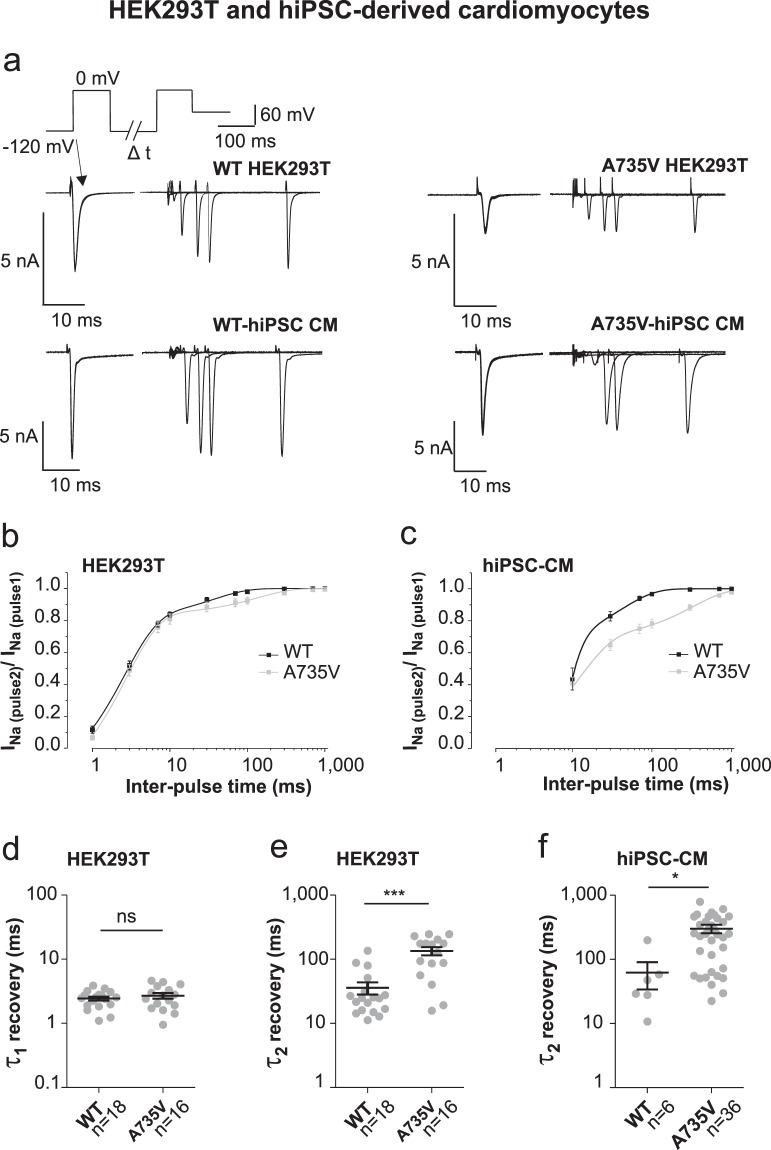
Mutation A735V prolongs the time constant of recovery from inactivation for Na_V_1.5 sodium channels expressed in hiPSC-CM and HEK293T cells. (**a**) Two pulse protocol to investigate the recovery from inactivation and representative current responses of WT and mutant A735V Na_V_1.5 channels expressed in HEK293T cells or hiPSC-CMs. Inter-pulse interval (Δt) varied from 1 to 1000 ms (inter-pulse voltage: -120 mV). (**b,c**) Time dependent increase of relative current amplitudes (I_Na (pulse2)_/I_Na (pulse1)_) fitted by a bi-exponential function reveal two time constants (τ_1_ and τ_2_) for recovery from inactivation of WT and mutant A735V Na_V_1.5 channels expressed in HEK293T cells (**b**) and hiPSC-CMs (**c**; data points < 10 ms are not displayed for hiPSC-CMs, according to improper fast voltage clamp conditions with large cardiomyocytes). (**d**–**f**) Scatter plot and mean values (±s.e.m.) of fast (**d**) and slow time constants (**e,f**) of recovery from inactivation for WT and mutant A735V channels expressed in HEK293T cells (**d,e**) or hiPSC-CMs (**f**). - Data volume in (b,d) and (e): WT, n = 18; A735V, n = 16. Data volume in (c) and (f): WT, n = 6; A735V, n = 36.

Even though the voltage dependence of inactivation seems less affected by the A735V mutation, the time dependence of recovery from inactivation might contribute to the reduction in sodium current amplitudes. Delayed recovery from inactivation could result in a reduced number of resting channels available for the next AP upstroke. Accordingly, we tested such properties in HEK293T cells and in hiPSC-CMs using a standard two-pulse protocol (Fig. 6a). Two activating voltage steps from −120 mV to 0 mV were successively applied, interrupted by an inter-pulse at −120 mV of varying durations between 0.01 and 1000 ms. The ratio of current amplitudes I_pulse2_/I_pulse1_ correlates with the recovery from inactivation and is plotted against the inter-pulse duration on a logarithmic x-scale in Fig. 6b,c. Data were best fitted by a bi-exponential function, yielding two time constants. In HEK293T cells, the first time constant is unaffected by the mutation (Fig. 6d; WT: τ1 = 2.4 ± 0.2 ms; A735V: τ1 = 2.7 ± 0.3 ms). However the second time constant is considerably prolonged by A735V as compared to WT (Fig. 6e; WT: τ2 = 36 ± 8 ms; A735V: τ2 = 135 ± 20 ms). A similar prolongation of the slow time constant was also observed in hiPSC-CMs (Fig. 6f; WT: τ2 = 62 ± 28 ms, n = 6; A735V: τ2 = 301 ± 46 ms). A fast time constant of recovery for hiPSC-CMs is not presented, since fast voltage clamp to −120 mV appeared insufficient in large hiPSC-CM cells, rendering results for very short inter-pulse durations unreliable.

## Discussion

The genetic profile of BrS is highly divergent (BrS type 1–19,^27^) and therapeutic attempts mainly focus on symptom treatment. Thus, specific models are needed to elucidate mutation-dependent molecular mechanisms causing the abnormal electrophysiological properties and pathologic electrocardiograms. To provide insights linked to Na_V_1.5 mutations, we applied genetic engineering at the hiPSC level followed by directed differentiation into predominantly ventricular-like CMs^20,21,28^. Different strategies have been used to promote hPSC-CMs maturation *in vitro*, including suspension aggregate- and monolayer-based approaches as well^13,16,29^. We here applied extended CM cultivation on a rigid surface to promote both maturation and uniformity of cells´ morphological and physiological properties^19^. Fast AP upstroke velocities in WT controls confirmed the proper expression of sodium channels. However, adequate expression of K_ir_ channels remains challenging, as indicated by the depolarized membrane potentials at rest. An elegant approach to circumvent this problem is the *in silico* injection of I_K1_ currents (yielding controllable resting membrane potentials around −80 mV), essential for sodium channel investigation and well-grounded evaluation of phase 0 depolarization^16,29^. In our study we adjusted resting potentials by continuous injection of a constant hyperpolarizing current, thereby providing the mandatory basis for valuable sodium currents and AP upstrokes.

Our main findings revealed firstly, that the A735V mutation induced a prominent reduction in cardiac AP upstroke velocity due to a reduced sodium inward current, notably without reduction in mRNA and protein expression of Na_V_1.5. Secondly, a rightward shift of the sodium activation curve and a prolonged recovery from inactivation was observed. This corroborates the importance of reliable model systems to analyse gating mechanisms for enabling the understanding of channelopathies in general and BrS Na_V_1.5 channel mutations in particular. Consequently, we provide strong evidence that our human iPSC-CM model is suitable to encompass the defect gating characteristic of the A735V-Na_V_1.5 mutation and its impact on the abnormal AP profile in BrS patients.

While others derived patient-specific hiPSC for modeling BrS^10,13,16,29^, we applied a genetic background-independent approach to investigate the impact of the A735V-Na_V_1.5 mutation, which seems to represent a potentially ethnicity-independent BrS causative^6^. In principle, the two experimental designs are complementary and enable to distinguish between compensatory effects to the sodium channel mutation, which might be helpful to identify modulators of the disease. Our approach thereby avoids heterogeneity, which may otherwise be induced in hiPSC models resulting from patients’ specific polymorphism patterns^30,31^. Taking advantage of the efficiency of the CRISPR/Cas9 gene editing technique we decided to investigate a homozygously engineered mutation A735V-Na_V_1.5, reflecting clinically relevant cases^7^.

It is worth highlighting that our approach resembles typical AP and sodium current characteristics of native cardiomyocytes. For evoked APs in WT CMs, we observed upstroke velocities of 120 mV/ms closely resembling those of mature ventricular heart muscle cells (183 mV/ms^14^). Moreover, sodium current densities of 279 pA/pF in WT CMs were comparable to ~300 pA/pF reported for a model of human epicardial-like ventricular cells^32^. The voltage for half maximal activation and inactivation in hiPSC-CMs also resembled those of their native counterparts (V_0.5_ native cardiomyocytes: activation −38.6 to −52 mV and inactivation −88 to −102 mV,^13^). Noteworthy, the reduction of upstroke velocities and sodium current densities induced by mutation A735V-Na_V_1.5 was reproducibly reflected throughout all independent hiPSC clones tested. This underscores that our overall experimental strategy overcomes the common hPSC clone- and differentiation/maturation-dependent variability demonstrating its robustness against undesired inter-experimental variations.

The hiPSC-CM approach aims at providing a native cell environment with (potentially) all auxiliary channel subunits and other cell type specific proteins being expressed, which may be involved in the disease-causing process. Accordingly, contrasting hiPSC-CM results with a heterologous expression system is informative to distinguish between primary effects on channel properties *versus* secondary effects modulated by the native cardiomyocyte environment. Additionally, such systems’ complementation helps to avoid misinterpretation of effects that may result from (e.g. patch clamp technology inherent) shortcomings of voltage control in the relatively large hiPSC-CMs. Comparing CMs *versus* HEK293T cells modulated by the same A735V-Na_V_1.5 mutation, we found drastically reduced current densities and a comparable rightward shift by ~20 mV of the sodium channel activation curve in both systems. A minor shift of +5 mV was previously reported for A735V-Na_V_1.5 in a *Xenopus* oocyte model^8^. However, here, we found a leftward shift of the inactivation curve only in hiPSC-CMs, whereas this effect of the mutation was neither observed in our HEK293T model nor previously indicated in *Xenopus* oocytes^8^. The four β-subunits (β1–4) of Na_V_1.5 are linked to the pore forming α-subunit and have been detected in hiPSC-CMs but not in HEK293T cells^33^. As the β_3_-subunit is thought to be upregulated in iPSC-CMs and its knockdown led to a leftward shift in inactivation curve^15^, its lack in HEK293T cells may explain the divergent results between these systems.

We also aimed at monitoring potential secondary effects that may have resulted from the A735V-Na_V_1.5 mutant on other ion channels. However, focusing on the inward calcium current that supports the plateau phase of the AP no relevant difference between WT and mutant hiPSC-CMs was observed (Supplementary Fig. S1). In BrS type 1, loss of Na_V_1.5 channel function was reported to coincide with a heterogeneity in the transient outward potassium current I_to_ resulting in a transmural electric gradient between the epi- and endocardium, in addition to a strongly reduced AP upstroke velocity^34^. Indeed, a direct influence of Kv4.3 channel expression (I_to_) on Na_V_1.5 current reduction has been recently postulated^35^. However, we could not identify any modification of I_to_ currents in relation to the A735V-Na_V_1.5 mutation tested here (Supplementary Fig. S2).

In summary, this study provides a comparative analysis of the A735V-Na_V_1.5 mutation in CRISPR/Cas9 engineered hiPSC-CMs and a human heterologous expression system to unravel direct pathophysiological effects of such BrS related mutation on AP characteristic and cardiac sodium channel physiology. Generating hiPSC-CMs with more physiological resting potentials was essential to identify a shifted activation curve, which represents a key mechanism underlying the strongly reduced upstroke velocities and abnormal BrS-related AP characteristics of mutation A735V. Testing CMs derived from several independently engineered hiPSC clones and numerous independent experimental repeats confirmed the validity and robustness of the data. This underscores that our novel hiPSC-CM model – in combination with the applied differentiation and maturation strategy – is highly relevant for investigating sodium channel mutations underlying the disease phenotype in BrS patients.

## Methods

### Induced pluripotent stem cell culture and generation of A735V-Na_V_1.5 mutated hiPSC lines

Mutation g.2204C > T in *SCN5A* encoding for A735V-Na_V_1.5 was introduced into hiPSCs by Clustered Regularly Interspaced Short Palindromic Repeats (CRISPR) with CRISPR-*associated* (Cas9) system^36,37^ and successful mutation was confirmed by DNA sequencing. Using 4D-Nucleofector (Lonza, Frederick) the resulting construct pSpCas9(BB)-2A-Puro (px459) V2.0 with sgRNA and the repair template were transfected into human iPSCs recently isolated from a male healthy subject’s dermal fibroblast (HDF-iPSC1 cells) with normal karyotype (46, XY)^38^. Briefly, iPSCs grown on Matrigel (corning) in mTeSR medium (Stem Cell Technologies) were treated with 10 μM Y-27632 (Sigma) for 1 hour before being dissociated into single cells with Accutase (Stem Cell Technologies). For one reaction in a strip, 500,000 hiPS cells were combined with 2 μg pSpCas9 plasmid and 1 μg repair template. Cells were nucleofected with program CB150 (optimized for hESC or hiPSC), and subsequently seeded onto pre-warmed mTeSR medium with 10 μM Y-27632 in a 24-well Matrigel coated plate. 3–5 days later cells were dissociated and plated in limiting dilution aiming at seeding single cells per well into 96-well plate for clonal expansion. Resulting clones were individually sequenced to identify those having the desired mutation. PCR amplification and sequencing (see Supplementary Methods).

### hiPSC-CMs cultivation, directed cardiac differentiation and maturation

Directed cardiac differentiation of hiPSC was performed by recently established protocols in suspension culture or in monolayer using chemical WNT pathway modulators^20–22^. For further analysis cells were dissociated by Collagenase B (1 mg/ml, Roche) treatment for 15–30 minutes at 37 °C. The CMs content resulting from differentiation was assessed by flow cytometry (see Supplementary Methods). For patch clamp analysis 2×10^4^ cells per well (12 well plate) were plated on fibronectin + 0.1% gelatine-coated, round glass coverslips (placed in each well) in plating medium (80% IMDM, Invitrogen; 20% fetal calf serum, HyClone;1 mM L-glutamine, 0.1 mM ß-mercaptoethanol, and 1% nonessential amino acid stock, all Invitrogen) supplemented with 10 µM Y-27623 (ROCK inhibitor) for the first 72 h. To induce recently described hiPSC-CM maturation via long-term cultures^19^ medium was changed to RPMI1640 supplemented with B27 plus insulin (Life Technologies) without Y-27623. Medium was replaced every 2–3 days before patch clamping at ~27–42 days post cell seeding.

### Immunofluorescence staining

For immunofluorescence staining (IF) cardiac aggregates derived by hiPSC differentiation in suspension culture were cryo-protected by incubation in saccharose solution (30% wt/vol in PBS) followed by embedding in Tissue-Tek, and incubation at −80 °C ahead of generating 10 µm thick cryo-sections on a cryotome (see details in^21^).

For IF staining cardiomyocytes were derived by enzymatic dissociation of cardiac aggregates and plated for >27 days before fixation and analysis. Aggregates sections or plated cells were fixed with 4% paraformaldehyde for 15 min at RT. After blocking/permeabilisation using Tris-buffered saline (5% donkey serum, 0.25% Triton X-100) cells were incubated with anti-sarcomeric α-actinin (1:800, EA53, Sigma-Aldrich), anti-myosin heavy chain beta (1:2000, NOQ7.5.4D, Sigma), anti-Na_V_1.5 (SCN5A, 1:200, ASC-005, alomone Labs) with and without pre-incubation with Na_V_1.5 blocking peptide (for 30 min at room temperature, 1:100, alomone Labs), respective isotype controls (DAKO), and detected using appropriate Cy2, Cy3-conjugated antibodies (1:200, Jackson ImmunoResearch). Nuclei were counterstained with DAPI and samples were analysed using the Axio Observer A1 (Zeiss).

IF staining and analysis of pluripotency associated markers OCT4 and SOX2 of genetically engineered hiPSC clones were performed equivalently using anti-human OCT4 (1:100, Sc-5279, Santa Cruz) and anti-human SOX2 (1:200, AMAB91307, Sigma).

### Transfection of HEK293T cells and mutagenesis

HEK293T cells were transiently transfected by standard calcium phosphate precipitation technique with 4 µg of pcDNA3.1 plasmids encoding for human Na_V_1.5 channels or mutant A735V-Na_V_1.5 channels, respectively. Mutation A735V was introduced by Quik Change site directed mutagenesis (Agilent Technologies) using the forward primer 5′ CAACACACTCTTCATGGTGCTGGAGCACTACAACA3′ and the reverse primer 3′TGTTGTAGTGCTCCAGCACCATGAAGAGTGTGTTG5′. Correct amino acid exchange was confirmed by sequencing. We generally co-transfected 2 µg pEGFP (#6085-1, Clontech) to identify successfully transfected cells by their fluorescence. Cells were incubated for 24 hours at 37 °C in DMEM medium (supplemented with 1% Penicillin/Streptomycin, 10% FBS and 2 mM L-Glutamine, Gibco, Thermo Fisher Scientific), before splitting into 60 mm dishes for subsequent patch clamp recordings. Experiments were carried out 4–24 hours after dissociation of cells.

### Electrophysiology

Long-term plated human iPSC-derived cardiomyocytes were used to record single cell APs by means of patch clamp technique in the current clamp mode and corresponding ionic currents in the whole-cell voltage clamp mode at room temperature (RT). RT greatly facilitates recording of sodium currents, since fast channel kinetics impede voltage clamp measurements at 37 °C. We studied APs and ionic currents in hiPSC-CMs under the same conditions and physiologic solutions to directly compare upstroke velocities with sodium current densities for the same single cell (see Fig. 4d). Cardiomyocytes were kept in extracellular Tyrode solution containing [mM]: 140 NaCl, 5.4 KCl, 1.8 CaCl_2_, 1 MgCl_2_, 10 HEPES, 10 Glucose, adjusted with NaOH to pH 7.4. Intracellular pipette solution contained [mM]: 120 K-Gluconate, 10 Na-Gluconate, 1 MgCl_2_, 10 HEPES, 10 EGTA/KOH, 3 Mg-ATP, pH adjusted to 7.2 by the use of KOH. Calculated sodium equilibrium potential was +67 mV. For patch clamp recordings of Na^+^ currents from transiently transfected HEK293T cells, cells were maintained in an extracellular solution containing [mM]: 140 NaCl, 3 KCl, 1 MgCl_2_, 1 CaCl_2_, 10 HEPES and 15 Glucose, pH 7.4 adjusted with 25% TMA-OH. Pipettes were filled with a solution containing [mM]: 100 CsCl, 30 NaCl, 1 EGTA, 10 HEPES, pH 7.4 adjusted with CsOH. Calculated sodium equilibrium potential was +39 mV. Micropipettes with a resistance of 2–5 MΩ were pulled from borosilicate glass capillaries (1.2 mm O.D. × 0.94 mm I.D. Harvard Apparatus, GC120TF-10, Holliston MA) by a Sutter Micropipette puller (Model P-97, Sutter Instruments, CA) and polished using a microforge (MF-900, Narishige). Whole cell capacitance and more than 80% of the series resistance were compensated by an analogue procedure. P/4 leak subtraction was used for experiments with HEK293T cells. Recordings were low-pass filtered at 5 kHz and sampled at 100 kHz using an Axopatch 200B amplifier and Axon Digidata 1550 (Molecular Device, Sunnyvale CA). Data was analysed using pClamp 10.5 software (Axon, Molecular Devices, Sunnyvale CA) and Origin 8 G (OriginLab, Northampton, MA). Voltage dependence of Na_V_1.5 channel activation was studied by 300 ms long voltage steps between −80 and +80 mV starting from a holding potential of −120 mV (200 ms) with inter-sweep durations of 5 s. Due to the abundance of hyperpolarization-activated channels hiPSC-CMs cells were held at −60 mV between the sweeps. For investigation of sodium channel inactivation holding potentials varied between −140 mV and +20 mV (200 ms) followed by a test voltage step to 0 mV (300 ms). Recovery from sodium inactivation was examined by application of two subsequent 100 ms long depolarizing voltage steps from −120 mV to 0 mV with varying inter-pulse times of 1–1000 ms at −120 mV.

### Western blotting

HEK293T cells that heterologously expressed WT or mutant Na_V_1.5 channels were lysed and samples of equal total protein amount (standard BCA-assay) were run on 10% SDS-PAGE. The electrophoresed proteins were transferred to nitrocellulose-membrane (#RPN303D, Amersham Hybond ECL, GE Healthcare Europe). Subsequently, the membrane was cut between the 75 kDa and 50 kDa band (Precision Plus Protein dual color marker #161-0374, BIO-RAD). The section with proteins of higher molecular weight (≥75 kDa) was incubated with a primary rabbit anti-Na_V_1.5 antibody (ASC-005, alomone Labs, 1:1000 in 1x TBST with 1% BSA), whereas the other membrane section (≤50 kDa) received a mouse anti-GAPDH antibody (A-3, sc-137179, Santa Cruz Biotechnology, 1:4000 in 1x TBST with 1% BSA). Goat anti-rabbit IgG HRP (#31463, Thermo Fisher, 1:20000 in 1xTBST with 1% BSA) or rabbit anti-mouse IgG HRP (#31455, Thermo Fisher; 1:25000 in 1x TBST with 1% BSA) were used as secondary antibody to visualize Na_V_1.5 and GAPDH by Super Signal^TM^ West Pico/Femto Luminol (Thermo Fisher) and Fusion imaging system (Vilber Lourmat).

### Biotinylation

Cell surface membrane insertion of Na_V_1.5-WT/A735V channels was investigated by use of hiPSC-CMs and transfected HEK293T cells, grown in 10 cm petri dishes. Biotinylation reaction was performed with 0.25 mg/ml biotin (EZ-link sulfo-NHS-SS-biotin, Thermo Fisher Scientific) per dish for 30 min at 4 °C and subsequently stopped with 10 mM glycine solution. Cell lysis was achieved by addition of RIPA buffer (100 mM NaCl, 20 mM HEPES, 1 mM Na-orthovanadate, 1 mM NaF, 1 mM EDTA, 1% NP-40, 1% desoxycholate, 1% SDS and 1% Protease inhibitor mix, pH 7.4). Biotin labelled proteins were purified by NeutrAvidin (High Capacity Agarose Resin, Thermo Fisher Scientific) affinity chromatography, eluted with 2x SDS sample buffer and loaded together with the total cell lysates on a 10% SDS-PAGE for western blotting.

### Statistics

Data are depicted as mean values ± s.e.m. and statistical significance was assessed with the unpaired two-tailed Student’s *t*-test or with ANOVA. Significance levels are indicated as “ns” (not significant), *p < 0.05, **p < 0.01 or ***p < 0.001. Statistics was performed in GraphPad PRISM version 7 (GraphPad Software, CA).

## Supplementary information

Supplementary Information

## Data Availability

The datasets generated during and/or analysed during the current study are available from the corresponding author on reasonable request.
