# Supplementary material for: Comparing human iPSC-cardiomyocytes versus HEK293T cells unveils disease-causing effects of Brugada mutation A735V of NaV1.5 sodium channels
Source: Sci Rep. 2019 Aug 1;9:11173. doi: 10.1038/s41598-019-47632-4 (PMC6673693; doi:10.1038/s41598-019-47632-4)
Supplement: Supplementary file 1 — Supplementary Information [file 41598_2019_47632_MOESM1_ESM.pdf]

Supplementary Information

Scientific Reports

Comparing human iPSC-cardiomyocytes *versus* HEK293T cells unveils disease-causing effects of Brugada mutation A735V of $Na_V1.5$ sodium channels

Jeanne de la Roche [1a], Paweorn Angsutararux [2a], Henning Kempf [3,4], Montira Janan [2], Emiliano Bolesani [3], Stefan Thiemann [1], Daniel Wojciechowski [1], Michelle Coffee [3], Annika Franke [3], Kristin Schwanke [3], Andreas Leffler [5], Sudjit Luanpitpong [2], Surapol Issaragrisil [2b]*, Martin Fischer [1b], Robert Zweigerdt [3b]**

*To whom correspondence may be addressed:
delaroche.jeanne@mh-hannover.de, surapol.iss@mahidol.ac.th, zweigerdt.robert@mh-hannover.de

hiPSC-derived cardiomyocytes

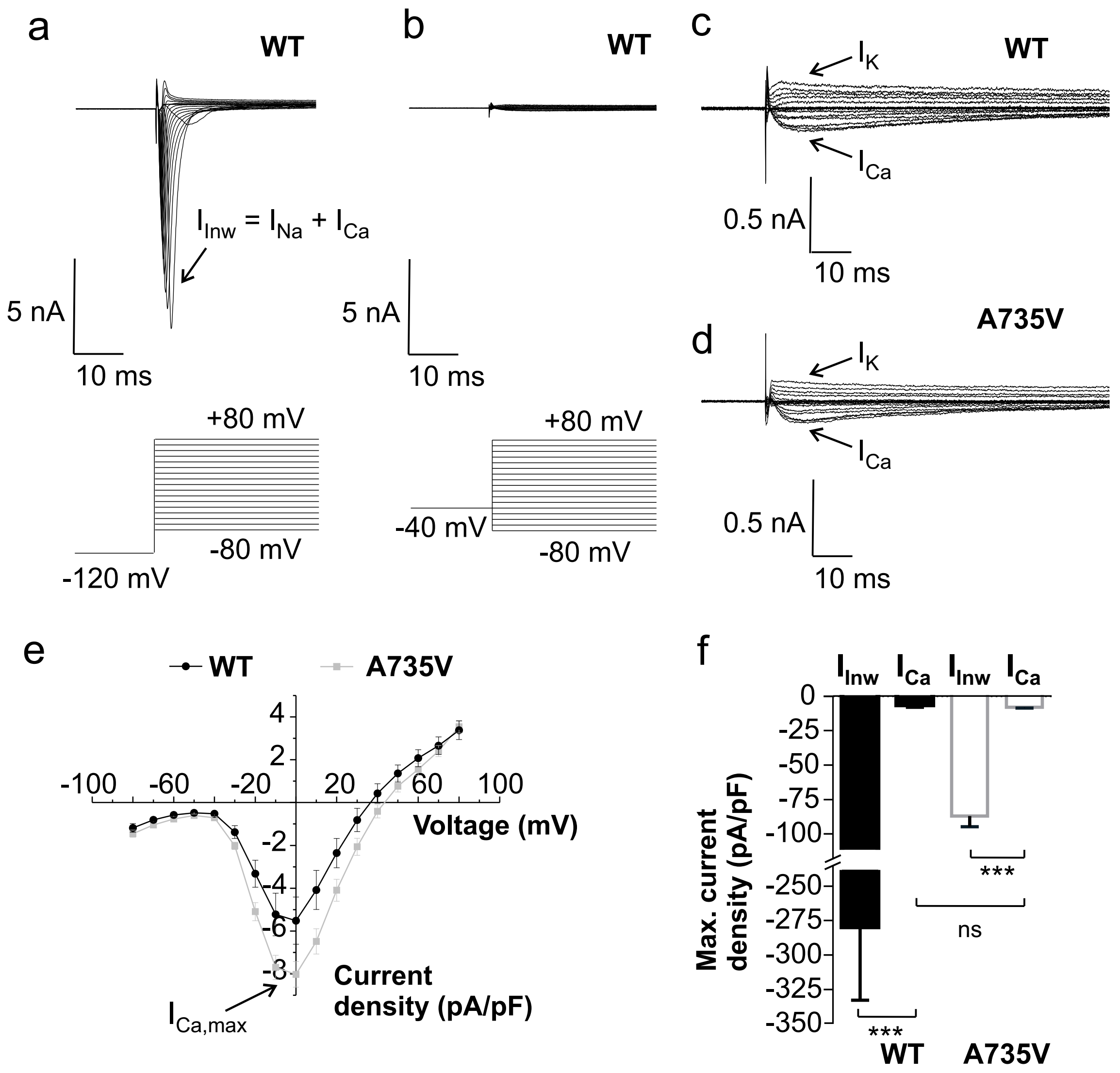

Supplementary Figure S1 *Determination of sodium currents in hiPSC-CMs by subtraction of calcium currents from total inward currents*

a) Representative recording of total inward currents (sum of sodium and calcium currents) from WT hiPSC-CMs elicited by a voltage protocol starting from a pre-pulse potential of -120 mV. b-c) Representative current trace starting from a pre-pulse potential of -40 mV, which inactivates sodium channels. Please note that current traces comprise calcium inward currents and potassium outward currents as displayed on an extended scale in (c). d) Comparable currents as in (c) obtained from an A735V hiPSC-CM. e) Voltage dependence of current densities between -80 and +80 mV for WT and A735V hiPSC-CMs. f) Comparison of maximum densities of total inward currents (at -40 mV for WT/ -10 mV for A735V, respectively) and calcium currents (at 0 mV) for WT and A735V hiPSC-CMs. – Data volume in (e) and (f): WT I_{Inw}, n = 20; WT I_{Ca}, n = 19 and A735V I_{Inw}, n = 57; A735V I_{Ca}, n = 57.

hiPSC-derived cardiomyocytes

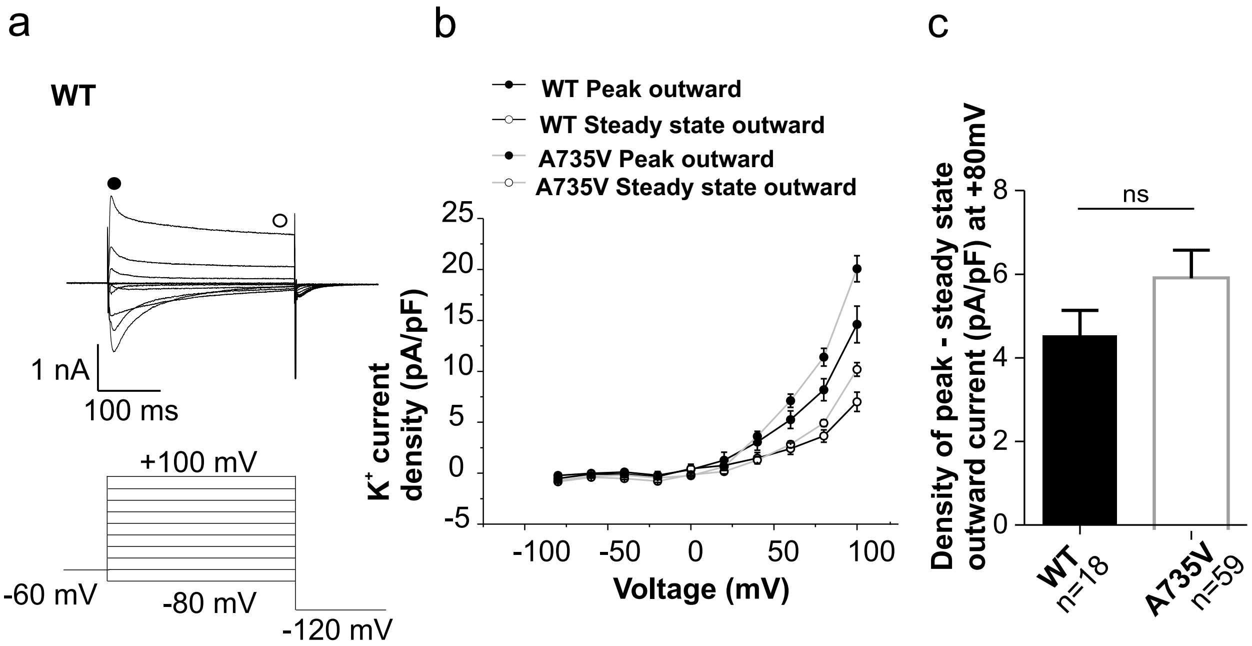

Supplementary Figure S2 *Potassium outward currents are unchanged by mutation A735V in hiPSC-CMs*

a) Representative current traces showing peak (●) and steady state (O) K^+ outward currents in WT hiPSC-CMs. The corresponding voltage protocol is depicted below. b) Voltage dependence of peak and steady state outward current densities for WT (black line) and A735V (grey line) hiPSC-CMs. c) Bar graph showing the relaxation from peak to steady state current densities that is mainly related to the transient potassium current (I_{to}). – Data volume in (b) and (c): WT, n = 18; A735V, n = 59.

HEK293T

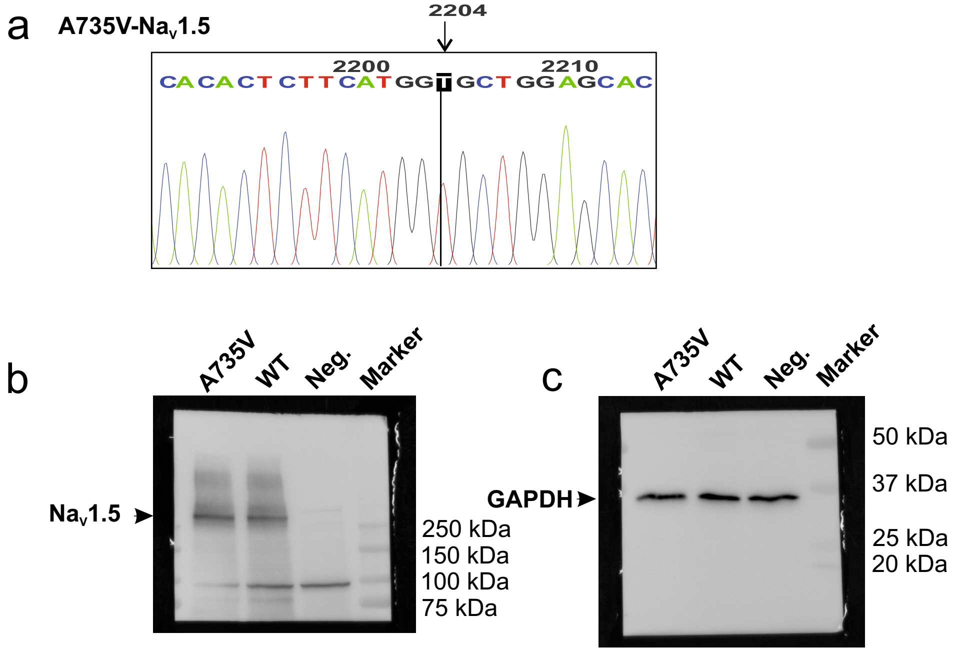

Supplementary Figure S3 *Sequence chromatogram for A735V-Na_v1.5 and its protein expression compared to WT in HEK293T cells*

a) Sequence section of A735V-Na_v1.5 cDNA with the corresponding point mutation introduced at position c.2204C>T. b) Representative full-length western blot for expression of WT and A735V-Na_v1.5 channels in transfected and untransfected (Neg.) HEK293T cells. Note, that there are two markers, the marker ahead of the samples is not labelled because only parts are transferred from the gel to the nitrocellulose-membrane (see left side of the blot). Na_v1.5 protein is visualized by anti-Na_v1.5 antibody (#ASC-005, alomone Labs) (Watanabe H *et al.* (2011) Circulation 124,1001). c) Representative full-lenght western blot for expression of GAPDH in transfected (WT and A735V- Na_v1.5) and untransfected (Neg.) HEK293T cells.

HEK293T

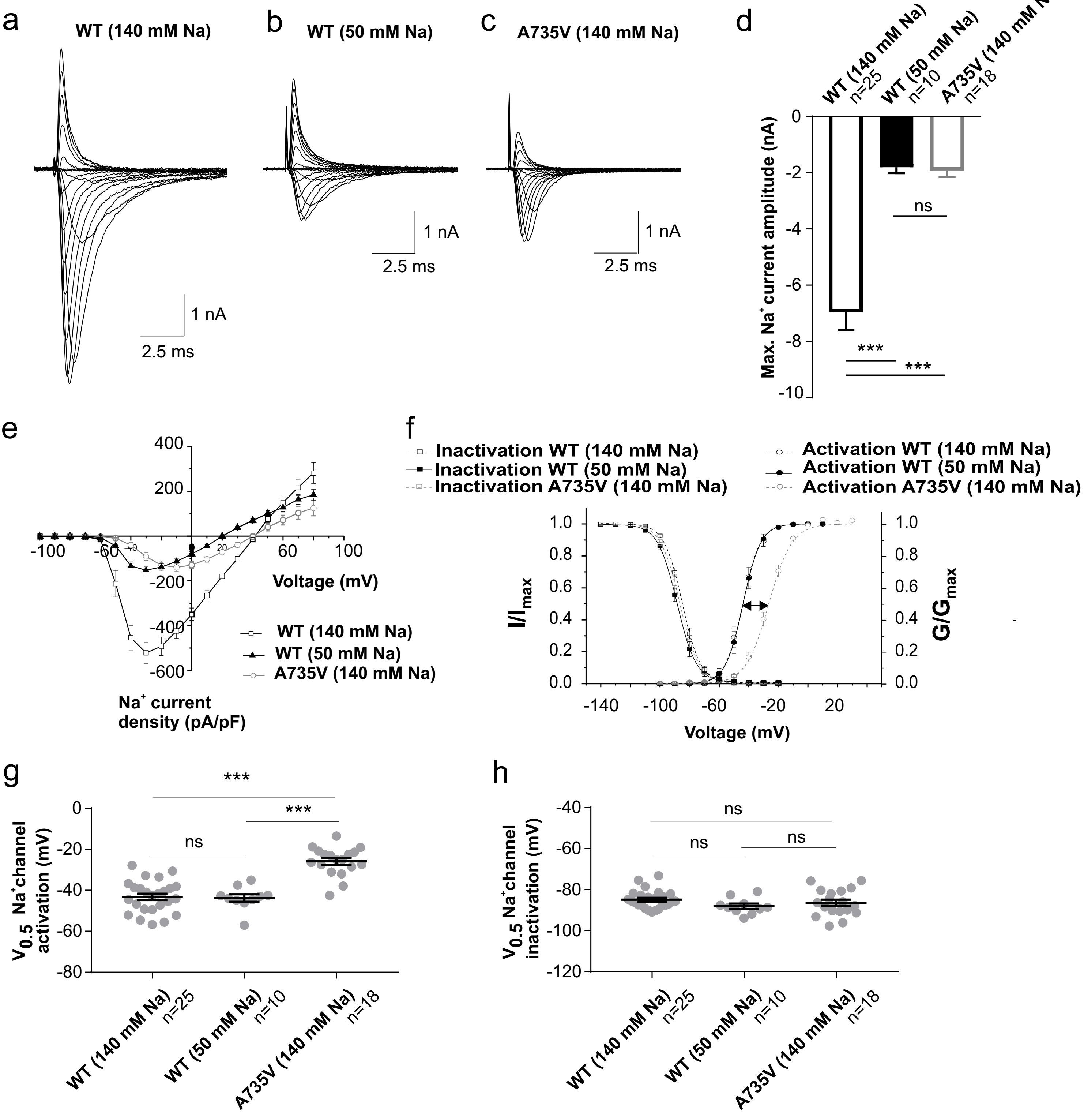

Supplementary Figure S4 *$Na_V1.5$ channel properties under high and low extracellular sodium concentration in HEK293T cells*

a-c) Representative current traces for WT and A735V-$Na_V1.5$ under physiological external sodium concentration (140 mM, a and c) and WT channels at low external sodium concentration (50 mM Na, b). d) Mean maximal sodium current amplitudes for WT (140 mM and 50 mM Na) and A735V (140 mM Na)-$Na_V1.5$ channels. Note, that WT maximal sodium current amplitudes appeared at -30 mV, whereas those of A735V were observed at -10 mV. e) I-V curves for the corresponding WT (140 mM and 50 mM Na) and mutant A735V (140 mM Na) sodium channels. f) Activation and inactivation curves for the three conditions as indicated. g-h) Scatter plots and mean values (± s.e.m.) of mid voltages ($V_{0.5}$) of activation (g) and inactivation (h) for WT (140 mM Na), WT (50 mM Na) and A735V (140 mM Na). - Data volume in (e) and (f): WT (140 mM Na), n=25; WT (50 mM Na), n=10 and A735V (140 mM Na), n=18.

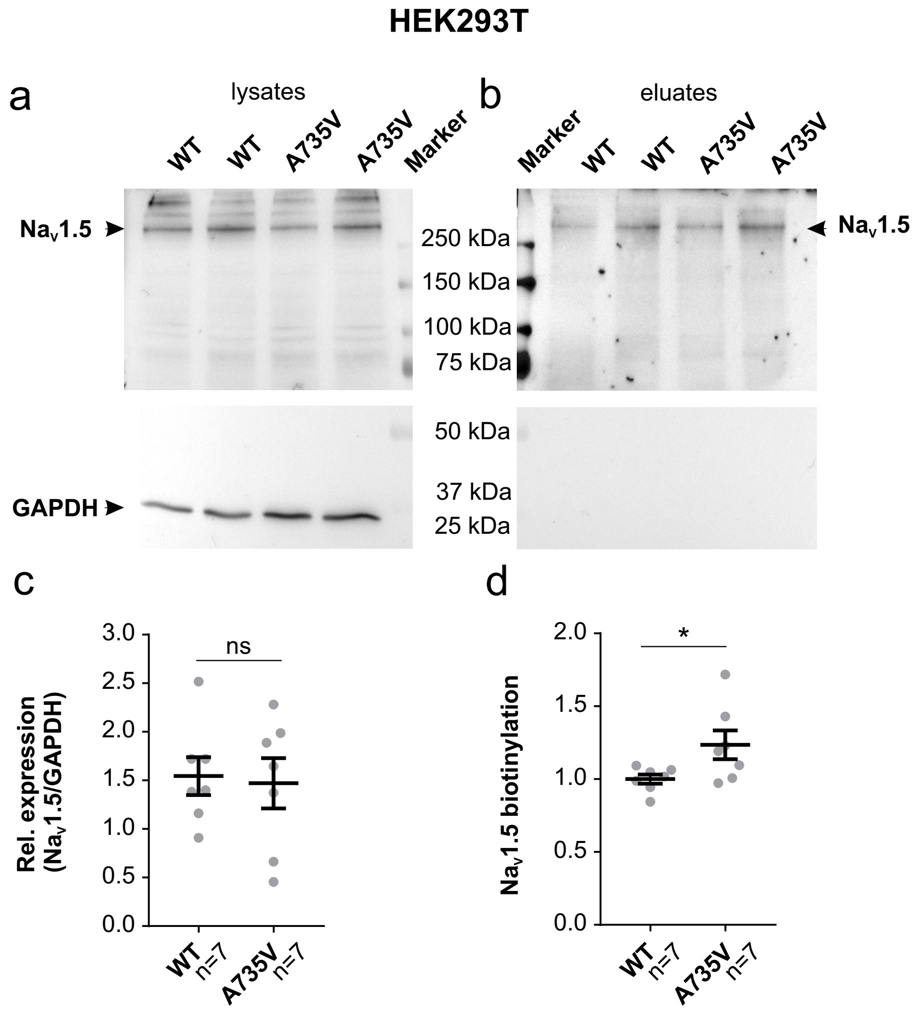

Supplementary Figure S5 *Protein expression and biotinylation of WT and A735V-Na_v1.5 channels in HEK293T cells*

a) Representative western blot for Na_v1.5 (upper membrane section) and GAPDH (lower membrane section) expression of whole cell lysates from WT and A735V-Na_v1.5 HEK293T cells. Analyzed protein bands for Na_v1.5 (above 250 kDa) are indicated by an arrow. Notably, additional bands above appear to represent protein aggregates or glycosylated channels as known for Na_v1.5 (Marionneau C *et al.* JMCC 2015). b) Representative western blot for biotinylated eluates from WT or A735V-Na_v1.5 expressing HEK293T cells after NeutrAvidin affinity chromatography. c) Scatter plot and mean values (± s.e.m.) for relative expression of Na_v1.5/GAPDH. d) Scatter plot and mean values (± s.e.m.) for the surface membrane inserted and biotininylated Na_v1.5 protein fraction (eluate/lysate). Values are normalized to the mean of WT-Na_v1.5.

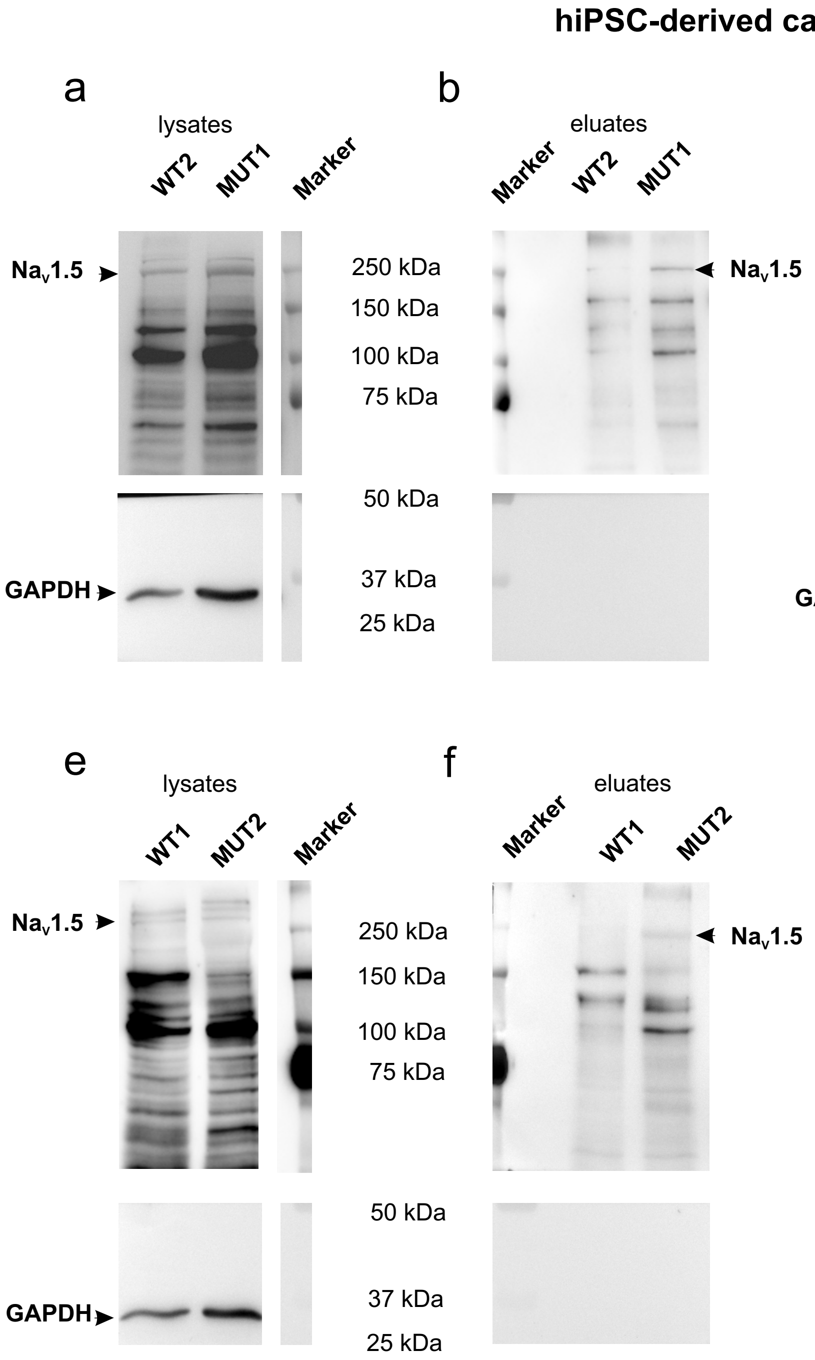

Supplementary Figure S6 *Protein expression and biotinylation of WT and A735V-$Na_v1.5$ channels in hiPSC-CMs*

a) Representative western blot for $Na_v1.5$ (upper membrane section) and GAPDH (lower membrane section) expression of whole cell lysates from WT and A735V-$Na_v1.5$ hiPSC-CMs. Analysed protein bands for $Na_v1.5$ and GAPDH are indicated by an arrow. b) Representative western blot for biotinylated eluates from WT and A735V-$Na_v1.5$ expressing hiPSC-CMs after NeutrAvidin affinity chromatography. c) Representative western blot for $Na_v1.5$ and GAPDH expression of whole cell lysates from WT and A735V-$Na_v1.5$ hiPSC-CMs after incubation with the $Na_v1.5$ block peptide. d) Representative western blot for biotinylated eluates from WT and A735V-$Na_v1.5$ expressing hiPSC-CMs after incubation with the $Na_v1.5$ block peptide. e-f) Comparable western blots for lysates (e) and eluates (f) as shown in (a-b) obtained from a second clone of WT and mutant $Na_v1.5$ hiPSC-CMs.

HEK293T

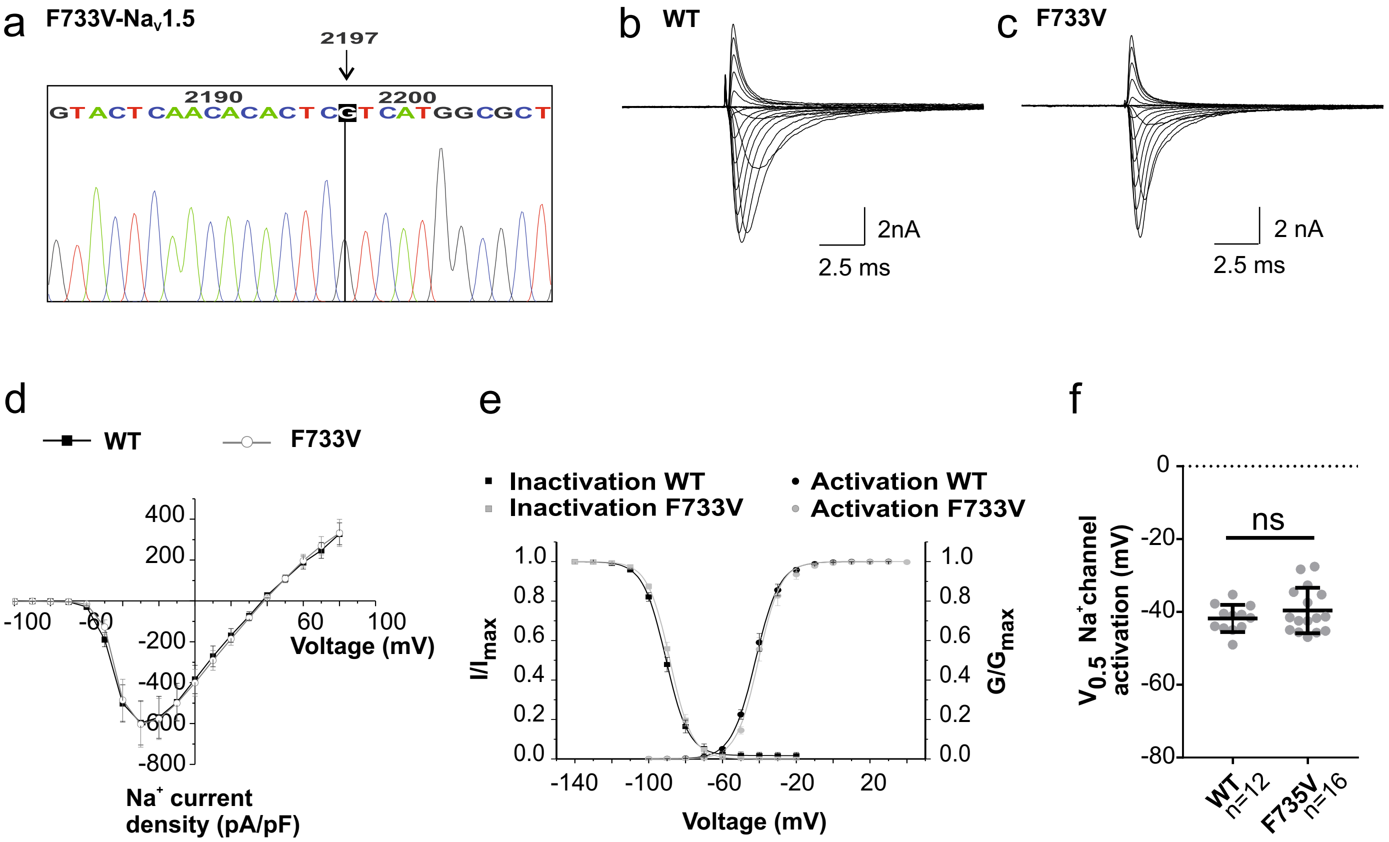

Supplementary Figure S7 *Sequence chromatogram for F733V-Na_V1.5 and its channel properties compared to WT in HEK293T cells*

a) Sequence section of F733V-Na_V1.5 cDNA with the corresponding point mutation introduced at position c.2197T>G. b-c) Representative current traces for WT and F733V-Na_V1.5 channels in transfected HEK293T cells. d) I-V curves for WT and F733V channels. e) Activation and inactivation curves for WT and F733V channels.
f) Mid voltages ($V_{0.5}$) of activation for WT and F733V channels. - Data volume in (d-e): WT, n=12 and F733V, n=16.

Supplementary Methods

Comparing human iPSC-cardiomyocytes *versus* HEK293T cells unveils disease-causing effects of Brugada mutation A735V of $Na_V1.5$ sodium channels

Jeanne de la Roche [1a], Paweorn Angsutararux [2a], Henning Kempf [3,4], Montira Janan [2], Emiliano Bolesani [3], Stefan Thiemann [1], Daniel Wojciechowski [1], Michelle Coffee [3], Annika Franke [3], Kristin Schwanke [3], Andreas Leffler [5], Sudjit Luanpitpong [2], Surapol Issaragrisil [2b]*, Martin Fischer [1b], Robert Zweigerdt [3b]**

*To whom correspondence may be addressed:
delaroche.jeanne@mh-hannover.de, surapol.iss@mahidol.ac.th, zweigerdt.robert@mh-hannover.de

Induced pluripotent stem cell culture and generation of A735V-Na_V1.5 mutated hiPSC lines

Plasmid pSpCas9(BB)-2A-Puro (px459) V2.0 (Addgene, Plasmid #62988), containing scaffold backbone for single-guide RNA (sgRNA) with hU6 promotor and Cas9, was used for genome editing. The 20-nt guide sequence (CAACACACTCTTCATGGCGC) was selected from online CRISPR Design Tool (http://crispr.mit.edu), to target the site of interest. Top and bottom strands of sgRNA oligonucleotides (5' caccGCAACACACTCTTCATGGCG 3' and 5' aaacCGCCATGAAGAGTGTGTTGC 3'; small letters indicate sequence used for cloning) were synthesized and were annealed by T4 polynucleotide kinase (T4 PNK) (New England Biolabs) in a thermocycler at 37 °C for 30 minutes, 95 °C for 5 minutes, and cooling down at 5 °C per minute to 25 °C. The annealed oligonucleotides were then cloned into plasmid pSpCas9(BB)-2A-Puro (px459) V2.0 using BbsI (New England Biolabs). The repair template was synthesised as single strand oligonucleotides (ssODN) including respective point mutation from C to T and two 80-nt homology arms.

PCR Amplification and sequencing

To monitor the CRISPR/Cas9 mediated modification, genomic DNA was extracted using Gentra Puregene Cell Kit (Qiagen), and stretches surrounding the target region where amplified by PCR using Q5 High-Fidelity DNA Polymerase (New England Biolabs) with primers as listed in Table 1. PCR products were purified with GenepHlow Gel/PCR Kit (Geneaid), and subjected to sequencing *via* GENEWIZ where the mutation is confirmed with CodonCode Aligner software.

Supplementary Table S1: List of primers for PCR

C2204T F-primer	5' CTAGCAGCCCTGTCATCTCC 3'
C2204T R-primer	5' GCAAGCTCCGTCCATAAGAG 3'

Flow cytometry

For quantitative flow cytometry analysis, dissociated cells at day 10-14 of differentiation were fixed and permeabilised (fix and perm kit, An der Grub) and incubated with primary antibodies against anti-sarcomere-specific pan-MyHC antibody (1:20, MF 20, Hybridoma Bank), anti-cardiac Troponin T (1:200, clone 13-11, Thermo Scientific), anti-sarcomeric α-actinin (1:800, EA53, Sigma-Aldrich) and respective anti-IgG isotype controls (DAKO), followed by an incubation with CyTM5-conjugated secondary antibody (1:200, Jackson ImmunoResearch). Data were acquired on an Accuri C6 flow cytometer (BD Biosciences) and analyzed using FlowJo software (Flowjo, LLC).

Analysis of mRNA expression by quantitative real-time PCR (qRT-PCR)

Total RNA was prepared using the RNeasy Kit (Macherey-Nagel) and reverse transcribed with Superscript II (Invitrogen) using oligo dT primers according to manufacturer’s instructions. Primer sequences are listed in Table 2. qRT-PCR was performed in duplicates using a Mastercycler® ep realplex^2 (Eppendorf). The size of amplicons and absence of nonspecific products was controlled by melting curves. Relative changes in gene expression were analysed using the Mastercycler® ep realplex Software Version 2.0 (Eppendorf). Expression levels of the target genes *SCN5A* and *MYH6* were normalised to β-actin (Fig. 1d).

Supplemenary Table S2: List of primers used for qRT-PCR

hu ß-Actin sense	5’ AGC CTC GCC TTT GCC GA 3’
hu ß-Actin reverse antisense	5’ CTG GTG CCT GGG GCG 3’
hu SCN5A sense	5' ATG GCA ATC CAC CCC AAG AG 3'
hu SCN5A reverse antisense	5' CCG GAA GAT GGT CTT TT 3'
hu MYH6 sense	5’ ACG ACT ACG CCT TCG TGT CT 3’
hu MYH6 reverse antisense	5’ GCT TGT AGA CGC CAG CTT TC 3’
